# The future of paediatric sleep medicine: a blueprint for advancing the field

**DOI:** 10.1111/jsr.14482

**Published:** 2025-03-28

**Authors:** Angelika A. Schlarb, Sarah Blunden, Serge Brand, Olivero Bruni, Penny Corkum, Rosemary S. C. Horne, Osman S. Ipsiroglu, Mirja Quante, Karen Spruyt, Judith Owens

**Affiliations:** ^1^ Faculty of Psychology and Sports Science, Department of Psychology Bielefeld University Bielefeld Germany; ^2^ Appleton Institute of Behavioural Science College of Health and Medical Sciences Wayville South Australia Australia; ^3^ School of Educational Psychology and Counselling, Faculty of Education Monash University Melbourne Victoria Australia; ^4^ Centre for Affective, Stress and Sleep Disorders (ZASS) Psychiatric Clinics of the University of Basel Basel Switzerland; ^5^ Faculty of Medicine, Department of Sport and Health Science, Sport Science Section University of Basel Basel Switzerland; ^6^ Kermanshah University of Medical Sciences (KUMS), Department of Psychiatry, Substance Abuse Prevention Research Center Health Institute Kermanshah Iran; ^7^ Department of Psychiatry, Sleep Disorders Research Center Kermanshah University of Medical Sciences (KUMS) Kermanshah Iran; ^8^ School of Medicine Tehran University of Medical Sciences Tehran Iran; ^9^ Center for Disaster Psychiatry and Disaster Psychology Center of Competence of Disaster Medicine of the Swiss Armed Forces Basel Switzerland; ^10^ Department of Developmental and Social Psychology Sapienza University Rome Italy; ^11^ Department Psychology & Neuroscience Dalhousie University Halifax Nova Scotia Canada; ^12^ Department of Paediatrics Monash University Melbourne Victoria Australia; ^13^ BCCH – Interdisciplinary Sleep Program & BCCH Research Institute Sleep/Wake‐Behaviours Lab, Department of Pediatrics University of British Columbia Vancouver British Columbia Canada; ^14^ Sleep Program Division of Child & Adolescent Psychiatry Vancouver British Columbia Canada; ^15^ Department of Neonatology University of Tuebingen Tuebingen Germany; ^16^ INSERM, NeuroDiderot Université Paris Cité Paris France; ^17^ Hospital Robert Debré AP‐HP Paris France; ^18^ Boston Children's Hospital Harvard Medical School Boston Massachusetts USA

**Keywords:** adolescents sleep, children sleep, diagnostics, sleep disorders, sleep health, treatment

## Abstract

Paediatric sleep medicine has rapidly evolved and expanded over the past half century as it became increasingly recognised as a unique field related to but distinct from adult sleep medicine. In looking forward to the next years, the focus of the following discussion is two‐fold: to summarise a brief history of the field, recent developments and current trends, and to present a blueprint for the future across various key domains. Using Bronfenbrenner's Ecological Systems Theory as a model for the interaction between the five interconnected ecosystems and sleep in children, we discuss a variety of topics relevant for the present state and future of paediatric sleep medicine. Such topics include the potential effects of climate change and war on children's sleep, the development of public policy initiatives—such as sleep education in schools and in communities, and global efforts to reduce the epidemic of insufficient sleep. Indeed, insufficient sleep contributes to a myriad of negative medical, mental health, functional, and safety consequences. We also focus on the development of paediatric sleep medicine‐specific educational initiatives and training programmes, and we showcase professional organisations such as the International Paediatric Sleep Association that are dedicated to the global expansion of paediatric sleep medicine. Finally, we address the need for further interdisciplinary collaborations, identify critical research gaps and explore the potential role of artificial intelligence and other new technologies in paediatric sleep research, including standardisation of sleep measurements, and novel methods of monitoring sleep in children.

## 1 INTRODUCTION

This present discussion provides the overview of paediatric sleep medicine. First, we showcase the short history of the paediatric sleep medicine; next, we describe its current situation, and last, we highlight possible future developments.

### 1.1 Where are we coming from? A brief history of paediatric sleep medicine

The study of sleep in infants, children and adolescents has undergone a significant evolution over the past century, parallel to the overall advancements in the science of sleep and chronobiology. Importantly, paediatric sleep medicine has emerged as a field related to, but distinct from adult sleep medicine. The early 20th century marked the beginning of a fundamental understanding of the architecture and regulation of sleep in humans, such as the identification and characterisation of non‐rapid eye movement (NREM) and rapid eye movement (REM), a sleep stage, which Aserinsky described for the first time in newborns (Aserinsky & Kleitman, 1953; Hobson, 1995). These early studies also began to highlight key differences in children's sleep architecture and sleep regulation: Compared to adults, children display increased slow‐wave sleep (SWS), more frequent nocturnal arousals due to shorter sleep cycles, the need for daytime sleep periods (naps) related to the more rapid accumulation of the sleep drive across periods of wake, and a general greater sleep need. In parallel, it turned out that standardised polysomnography (PSG) assessments for adults were not appropriate for newborns and young children. For example, building upon the early work of Kales et al. (1968) and other researchers, in the 1970s Dr Parmalee, Anders and Guilleminault were among the pioneers for scoring electroencephalographically assessed brain wave oscillations into meaningful sequences of sleep stages in infants and children (Parmelee et al., 1968).

Paediatric sleep medicine as a unique clinical field was first acknowledged in the 1960s and 1970s; researchers observed child‐specific sleep characteristics, including their risk factors and treatment regimen, such as parasomnias, nocturnal enuresis and obstructive sleep apnea (OSA) (Guilleminault et al., 1972; Guilleminault et al., 1976). This period marked the growing recognition of the clinical significance of sleep disorders in paediatric populations. For example, in 1963, Gastaut and Broughton hypothesised that most ‘episodic phenomena during sleep’ in children were non‐epileptic in nature and occurred out of NREM sleep, predominantly out of SWS. In 1968, Broughton termed such events ‘disorders of arousal’ (Broughton, 1968), and subsequently such events were labelled as the partial arousal parasomnia disorders of confusional arousals, sleepwalking and sleep terrors. The 1980s witnessed the establishment of paediatric sleep clinics and the use of PSG as a diagnostic tool, underscoring the impact of sleep disorders on daytime functioning, school performance, and overall development. In the 1990s, research expanded to include behavioural sleep problems, contributing to the understanding of bedtime routines and healthy sleep practices in promoting healthy sleep in children (Durand & Mindell, 1990; France & Hudson, 1990; Richman et al., 1985). The early 21st century saw an increased focus on the relationship between sleep and neurodevelopmental issues, psychosocial functioning, and the bi‐directional links between sleep disturbances and mental health conditions. In addition, potential long‐term adverse effects of sleep disordered breathing (SDB) in children on cardiovascular functions were identified. Cardiovascular issues were for instance high blood pressure, metabolic issues including type 2 diabetes, systemic inflammation, endothelial dysfunction, changes in autonomic regulation related to chronic intermittent hypoxia, gas‐exchange abnormalities. Importantly, such sleep‐related cardiovascular issues were also related to cognitive and emotional issues. Finally, there is increasing recognition of the bi‐directional relationship between sleep and poor health outcomes such as childhood obesity, accidental injuries, and depression and suicidal behaviour. The emerging concept of paediatric sleep health in the last several decades has also allowed the identification of possible risk factors for poor sleep health at three levels: the individual (e.g., insufficient sleep opportunity, excessive screen time), the community (e.g., neighbourhood noise and light levels, pollution, early school start times) and national/international level (e.g., climate change, homelessness). All three levels have consequences both for the children's long‐term health and for policy makers and stake holders.

In summary, paediatric sleep medicine has evolved into an important subfield of both sleep medicine and paediatrics, and it showed that sleep disorders and poor physical and mental development are highly associated. With new scientific insights and technological advancements, paediatric sleep medicine is poised for a promising future.

### 1.2 Where are we now? Current state‐of‐the‐field of paediatric sleep medicine

Now, we know that the paediatric sleep medicine is a field related to, but distinct from adult sleep medicine. As such, paediatric sleep medicine needs specific modifications for clinical practice, research methodology, educational approaches and public policy unique to children, adolescents and their families. Aside from both specific electroencephalography (EEG) patterns and a characteristic stage‐by‐stage development (Scholle et al., 2011), common age‐ and population‐related topics include children's and adolescents’ sleep patterns, schedules, and behaviours. Factors associated to such patterns are family functioning, social media and screen usage, obesity, and physical activity, and the sleep‐related factors are additionally associated with academic achievement, mood, behavioural dysregulation, and daytime sleepiness. In the same vein, one such further factor is the impact of technology on children's sleep. Studies on the screen–sleep link recommended limiting the screen exposure before sleep and during the night for children and adolescents. Other studies have focused on the sleep–mental health link and on possible treatments. Further emerging topics included children's sleep in the context of natural disasters such as viruses, wildfires, and climate change, and in the context of man‐induced disasters such as war, displacement, refugee status, and terroristic attacks.

In the following section, we highlight some of the most impactful developments in paediatric sleep medicine over the past several decades. Such developments help to understand that paediatric sleep medicine needs further research, including educational programmes and continuous efforts to improve children's sleep.

### 1.3 Recent developments

- *Sleep disorders and their associations*

1. Increased attention to paediatric sleep disorders and treatment: sleep disorders in children, such as sleep apnea, insomnia and restless legs syndrome (RLS), are increasingly recognised as serious health issues. Previous studies reported that approximately 25–30% of children experience significant sleep concerns at some point during childhood (Trosman & Ivanenko, 2021). Data from more recent studies suggest that the prevalence of childhood sleep disorders (e.g., SDB, insomnia) may be increasing both due to heightened awareness and recognition by caregivers and healthcare providers and to the presence of mounting risk factors (e.g., obesity, mental health and neurodevelopmental disorders such as autism). These prevalence estimates clearly demonstrate the urgent need for specialised, age‐specific and sleep‐related treatment approaches.
2. Differentiation and awareness of specific types of sleep disorders: both physiological and behavioural/psychological causes may confer to a broad variety of sleep disorders. In addition, specific sleep disorders primarily observed in childhood (e.g., head‐banging/body rocking, night‐time fears, partial arousal parasomnias, enuresis) may not cross into adolescence and adult life, while other sleep disorders (e.g., insomnia, narcolepsy, and central hypersomnias) get more prevalent in adolescence and beyond.
3. Link between sleep, mental health and academic/school‐related performance: there is a growing understanding of the sleep–mental health–academic performance link, including attendance and dropout and graduation rates. Sleep deprivation and disrupted sleep are associated with a broad variety of mental health issues, including symptoms of attention deficit hyperactivity disorder (ADHD), anxiety disorders, depression, aggression, risk taking behaviour, poor family functioning, and neurobehavioral disturbances (Morales‐Muñoz & Gregory, 2023; Spruyt, 2019). Such results highlight the necessity for a holistic approach in paediatric sleep medicine.
4. Link between sleep and physical health: over a third of children complain about poor sleep; accordingly, there is a growing literature on the association with and the impact of poor sleep on daytime functioning and physical health. Typical targets are: growth (i.e., growth hormone secretion), immune function (i.e., sleep loss inducing inflammation), metabolic regulation (i.e., increased risk of obesity), and cardiovascular health (i.e., higher blood pressure and heart rate variability). Sleep‐related physical health issues are further associated with traffic and sports injuries, lower quality of life, and lower scores for mood, cognition and behaviour. Importantly, poverty, ethnic status and SDB may further substantiate the poor sleep–poor physical health link.
5. Increased attention to paediatric populations with special needs: there is a broader knowledge about the necessity of screening sleep disorders in children who are not typically developing such as those with neurodevelopmental disorders (Bruni et al., 2025) (e.g., autism spectrum disorder), genetic disorders (e.g., Down syndrome), or those with other risk factors (e.g., prematurely born babies). Often, parents believe that their child's poor sleep is simply a feature of such specific health conditions. However, for instance the treatment of sleep disorders such as OSA can improve sleep and the quality of life of both the children and their families. Children with autism spectrum disorder have a high frequency, severity and persistence of sleep problems (up to 80% in some studies) (Belli et al., 2022; Fauroux et al., 2024). Further, the increasing prevalence rates of neurodevelopmental disorders implies a more systematic evaluation, diagnosis and management of sleep disorders.

B *Diagnostics and technological advances*

1. Development of age‐appropriate subjective sleep diagnostic measures: in recent years, several milestones concerning the diagnosis of childhood sleep disorders should be noted. In addition to specialised sleep logs appropriate for various ages, several paediatric screening and diagnostic tests were developed and validated like the Brief Infant Screening Questionnaire (BISQ; Sadeh, 2004), the Sleep Disturbance Scale for Children (SDSC; Bruni et al., 1996), the Children's Sleep Habits Questionnaire (CSHQ, Owens et al., 2000), and the BEARS (B = Bedtime issues, E = Excessive daytime sleepiness, A = night Awakenings, R = Regularity and duration of sleep, S = Snoring) screening tool (Owens & Dalzell, 2005), and the Patient‐Reported Outcomes Measurement Information System (PROMIS) Sleep Disturbance and Sleep Impairment Surveys (in both four‐ and eight‐item and caregiver and self‐report versions) are currently in widespread use both for research and clinical purposes (Forrest et al., 2018). In addition, there are a number of more targeted questionnaires such as the RLS drawings (DelRosso et al., 2024; Picchietti et al., 2013), the Childrens Sleep Comic (CSC), (Schwerdtle et al., 2016), or tests like the cataplexy test (Vandi et al., 2019).
2. Polysomnography: in‐laboratory paediatric PSG remains the ‘gold standard’ for diagnosing the severity of SDB, sleep‐related movement disorders (i.e., periodic limb movements), some partial arousal parasomnias and unexplained daytime sleepiness. PSG represents a very detailed single night ‘sleep snapshot’ that includes EEG, electro‐oculography and electromyography for evaluating sleep architecture; respiratory parameters including respiratory effort, obstructed breathing and oxygenation/ventilation; heart rate, and body movements. It should also be noted that mandatory training and certification of ‘adult’ sleep laboratories that have expanded to include children should be considered in order to ensure quality control and appropriate monitoring, recording, scoring, and interpretation of paediatric studies. Finally, the use and validation of various home‐based ambulatory monitoring systems in children is still evolving but represents an important potential solution to the relative lack of access to paediatric sleep laboratories in many areas of the world.
3. ‘Wearable’ sleep monitors: in contrast to PSG, sleep logs and activity monitors, including actigraphs, and advanced commercial wearables (such as one‐to‐three‐channel EEG devices), are designed to produce continuous data regarding night‐to‐night variability in sleep and sleep patterns in the natural home environment over time, and are most often used to diagnose and evaluate treatment of insomnia and circadian rhythm disorders (Guillodo et al., 2020).
4. Adaptations for at home usage: use of overnight oximetry is also routinely used by many sleep laboratories to screen children for SDB, so that those children identified with severe clusters of desaturation can be prioritised for sleep studies and or adenotonsillectomy and decrease the time taken before treatment (Wilson et al., 2024). Trials have also shown that PSG can be successfully conducted in the home of children (Ioan et al., 2020) and Level 2 studies in the home have also been shown to be technically successful and have excellent family acceptability (Griffiths et al., 2022). The use of other devices such as mattresses to screen for SDB also shows promise and may be particularly useful in children with neurodisabilities who require frequent sleep studies but who find attendance at a sleep laboratory particularly distressing (Collaro et al., 2023). New devices are being developed all the time for screening of SDB in adults and these also show promise as screening tools in children, but further research to validate these is required (Panichapat et al., 2024).

C *Age‐oriented prevention and intervention concepts*

1. Sleep books to educate families with children: experts in sleep have developed several sleep‐related books. For example the *Boss of My Sleep* book (Blunden, 2017), which is also available as ebook, *What to Do When You Dread Your Bed* (Huebner & Matthews, 2008), *Je dors donc je grandis!: Cerveau, croissance, apprentissages… Tous les secrets du sommeil* (Filliozat, 2020), *Ilvy Sleeps Well/Ilvy Schläft Gut* book, or for older children *Genial in Sleep/Genial im Schlaf* (Hödlmoser et al., 2019; Hödlmoser et al., 2021) just to name a few. However, caution should be exercised in making recommendations for families of bedtime books with sleep in the title, as not all endorse useful strategies for children to fall and remain asleep and may not always be culturally appropriate (Schlarb et al., 2021).
2. School‐based sleep health education: as sleep is also an important issue for school success, sleep and sleep hygiene educational programmes have been developed for school‐aged children and adolescents (Chung et al., 2017; Das‐Friebel et al., 2019; Rigney et al., 2021; Trindade & Ramos, 2020). The advantage of such programmes is that they can reach a larger number of children and adolescents and can impact family and school communities more broadly. Sleep education programmes in schools are diverse and aim to either increase sleep knowledge, or improve sleep quality and quantity or both, and various reviews have been published in the recent years (Blunden et al., 2012; Blunden & Rigney, 2015; Chung et al., 2017; Gruber, 2017).
3. Novel treatment approaches of various sleep disorders: for sleep disorders a variety of treatment approaches, platforms and delivery formats for a range of age groups have been developed during the last few decades. In particular, the coronavirus disease 2019 (COVID‐19) pandemic accelerated the acceptance and use of telemedicine, improving access to specialised services, particularly in rural or underserved areas (Sharma et al., 2021). Telemedicine solutions could facilitate continuous care and adjustment of treatment plans. In addition, face‐to‐face single treatment interventions and group treatments, as well as digital application (app)‐based interventions have enormous potential to increase access to and adherence with insomnia management (Bourchtein et al., 2020; Scantlebury et al., 2018), which consists of face‐to‐face single treatment interventions, group treatments, internet‐based treatments, or online treatments. Besides, sleep apps or digital intervention like *Better Nights Better Days* or the *Sleep Ninja* were developed to help children and adolescents with sleep disorders (mainly insomnia disorders) gain increased attention (Corkum et al., 2018; Subotic‐Kerry et al., 2023).
  While behavioural interventions such as cognitive behavioural therapy for insomnia (CBT‐I) remain the mainstay and first choice for treating insomnia in children as well as adults, both over‐the‐counter (OTC) and prescription drugs are increasingly used by both caregivers and healthcare providers as the primary treatment modality for paediatric insomnia. There are many reasons for this trend, including relative lack of training of paediatric providers in employing behavioural strategies, as well as limited time to deliver these interventions in clinical practice; the preference of some caregivers for a ‘quick solution’, the ready availability in some countries of OTC sleep aides—such as sedating antihistamines and melatonin, and aggressive marketing by pharmaceutical companies.

D *Scientific developments*

1. High‐level publications: there has been a substantial increase in paediatric sleep medicine peer‐reviewed and PubMed‐listed publications over the past 20 years, e.g, >50,000 publications on sleep in children are currently listed in PubMed. Not only the number of publications but also publications in high impact journals were elevated.
2. Special associations and sections in professional sleep communities: the International Paediatric Sleep Association (IPSA) was founded in 2005 during the World Association of Sleep Medicine (WASM) meeting in Berlin, with the crucial contribution of Christian Guilleminault and Oliviero Bruni. The IPSA has held bi‐annual international paediatric sleep congresses since 2010. This is a society that is by nature interdisciplinary, as paediatric sleep spans a wide scope of disciplines (including paediatrics, neonatology, pulmonary, neurology, psychiatry, developmental medicine, and psychology). In addition, various specialised paediatric sections in sleep societies in various countries were initiated, e.g., the working group paediatric sleep medicine in Germany which has >300 members. Similarly, although in a much smaller in scale, experts in the field of neurology, paediatrics, and sleep medicine are highly engaged in the subsection of paediatric sleep medicine of the Swiss Society for Sleep Research, Sleep Medicine and Chronobiology (SSSSC) and the Australian Sleep Association and several others in different countries. After 52 years, the European Sleep Research Society established the first specialised paediatric sleep training programme. Due to its success and the growing demand for paediatric sleep education, the World Sleep Society now offers several paediatric sleep courses (Spruyt et al., 2024).

Despite these significant advances in paediatric sleep medicine, there is much work to be done. Therefore, in the next section, future development possibilities are described.

### 1.4 Where are we going? – Looking to the future

**FIGURE 1:**
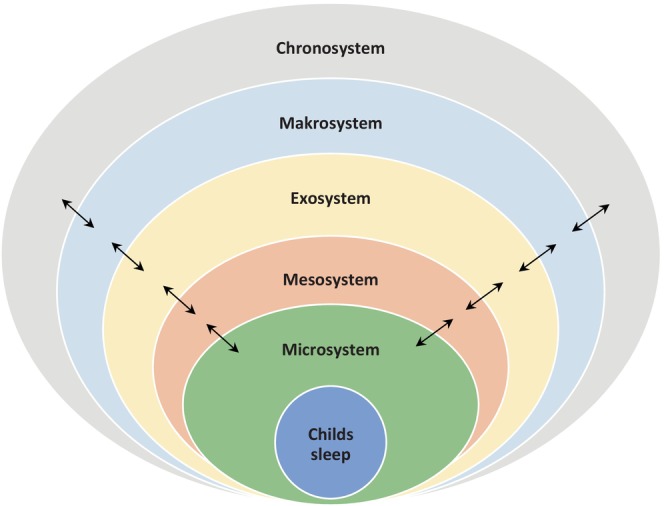
Future developments and research should implement all the facets of the sleep‐related Bronfenbrenner model.

To structure this essential part, we took the model of Bronfenbrenner (1986) (Figure 1; Alaribe et al., 2021). Bronfenbrenner's Ecological Systems Theory (Bronfenbrenner, 1986) posits that an individual's development (in our example sleep) is influenced by a series of interconnected environmental systems, ranging from the immediate surroundings (e.g., family) to broad societal structures (e.g., culture). The model includes the microsystem, mesosystem, exosystem, macrosystem, and chronosystem, each representing different levels of environmental influences on an individual's growth and behaviour. In addition to the model, we added other aspects like technical developments and other themes concerning future developments.

## 2 CHRONOSYSTEM FACTORS

### 2.1 Environmental changes/ potential effects of global warming

Global warming and climate change may significantly impact children's sleep. Higher temperatures and increased heat waves may disrupt sleep, as elevated temperatures may negatively affect sleep quality and duration. Obradovich et al. (2017) used data of 765,000 adult United States participants, from 2002 to 2011, and showed that warmer night‐time temperatures were associated with a higher prevalence of sleep disturbances. Risk factors were families with lower income and elderly adults. Importantly, children are particularly vulnerable to temperature changes due to their lower capacity for thermoregulation (Bach et al., 2011).

Additionally, climate change‐related environmental changes can exacerbate factors that already impact sleep due to geographical location such as increased air, light and noise pollution, or differences in heat and humidity closer to the equator. Children living in urban areas may be especially affected due to higher exposure to noise and poor air quality, which in turn appears to be associated with a higher risk of asthma and OSA (Spruyt et al., 2014).

Concerning improving sleep for children and adolescents confronted with global warming, ongoing and future research might focus on specialised mattresses, room climatisation, and other innovative solutions.

1. Specialised mattresses with thermoregulating materials. Specialised mattresses not only for adults but also for children and adolescents incorporating thermoregulating materials might help maintain a comfortable sleep temperature. Special materials might be able to absorb, store, and release heat to keep the sleeping surface within an optimal temperature range. Special fabrics may help evaporating sweat and keeping the sleeper dry, which is crucial for maintaining comfort during hot nights.
2. Adapted air conditioning systems and room climatisation. Modern air conditioning systems for homes with smart controls can optimise room temperature for sleep. Adapted and personal‐oriented systems might be able to adjust temperatures gradually to signal the body that it is time to sleep. Besides those aspects also light dimming and calming sound music or bedtime stories for children might be part of such systems.
3. Sleepwear and bedding innovations. Innovative sleepwear and bedding materials such as those infused with cooling fibres or moisture‐wicking technology can complement mattress and room climatisation efforts. For example, sleepwear with special preferred odour might help children to fall asleep easier. However, children's special requirements need to be considered such as age‐appropriate sleep clothing for infants to reduce the risk of sudden infant death syndrome.

### 2.2 War and natural disasters

Disasters are the result of exposure to a hazard that threatens personal safety and disrupts both community and family structures. Disasters lead to personal and societal loss, and disaster aftermaths usually exceed personal and community resources. Typically, disasters are clustered into naturally occurring disasters such as earthquakes, floods, wild fires, tornados, hurricanes, tsunamis, and man‐made disasters, which are further split into man‐made disasters with no deliberate intention of harm such as break‐downs of nuclear power stations, aeroplane and train crashes, shipwrecks and similar, and human‐made disasters with deliberate intention of harm such as wars, school shootings, or terrorist attacks (Everly & Lating, 2022; Ursano et al., 2017). Disasters impose significant stress on adults, in general, and on children and adolescents, in particular. Unsurprisingly, children's sleep is impaired in such a context (Ge et al., 2020; Lai et al., 2020; Zhang et al., 2015). Given this background, recommendations and supportive strategies should be developed to improve children's sleep and psychological functioning. Besides healthy and safe sleep recommendations, also age‐oriented materials, bedtime stories and further materials might both help children to handle such difficult situations better and to prevent the emergence of symptoms posttraumatic stress disorder (Sadeh et al., 2008).

## 3 MACROSYSTEM FACTORS

### 3.1 Sleep in professional education regarding children's health

Not only paediatricians, but psychologists, midwives, community nurses, pedagogues and other healthcare professions should have the topics of paediatric sleep patterns, sleep development, and sleep disorders, including sleep‐related comorbidity and treatment possibilities in their education programme and syllabus (Gruber et al., 2017.; Hulst et al., 2020; Richardson et al., 2021). This means that both university and education courses should include sleep issues and implement this important topic in their curricula. However, as noted above, up to date, childhood sleep and treatment of disorders appear to be neglected in the context of professional paediatric education (Spruyt et al., 2024).

### 3.2 Paediatric sleep coaches

As various studies have shown, in addition to board certified paediatric behavioural sleep experts, there are also a large number of infant and paediatric sleep coaches who market their services to caregivers. However, their education regarding a basic medical or health‐based profession is often not given. In addition, age‐related sleep diagnostics, knowledge of all the various sleep disorders in childhood and adolescence, treatment possibilities and comorbidity or risk factors for further impairments are often missing. Qualifications are needed and a structured certification process such as that required for lactation consultants through the International Consultant Lactation Association for paediatric sleep coaches might be a future goal to ensure quality sleep care for young children (Ingram et al., 2018).

### 3.3 Cultural differences/factors

Healthy sleep behaviour is perceived differently between countries and cultures. Western Educated, Industrialised, Rich and Democratic (WEIRD) societies (Henrich et al., 2010) tend to favour minimising parental assistance at bed‐time and after overnight wakings, to facilitate independent initiation and re‐initiation of sleep (D'Souza et al., 2024). However, in other countries and cultures, and even in some families within the same culture, withholding parental assistance is considered. Co‐sleeping (parent‐infant bed‐sharing) is often discouraged in WEIRD societies but more common in Asian and South American countries. Research indicates that paediatric sleep patterns, including sleep duration and quality, often vary by race, ethnicity, and cultural backgrounds. For instance, studies have shown that children from Hispanic and African American communities may experience more sleep disruptions, including shorter sleep duration and higher rates of sleep disorders, compared to Caucasian children. These early differences in paediatric sleep can set the stage for continued disparities in sleep health as individuals transition into adulthood, with higher rates of sleep apnea, insomnia, and other sleep disorders noted in these populations later in life (Fuller‐Rowell et al., 2017; Lin et al., 2019; Singh et al., 2024).

### 3.4 Sleep‐friendly hospitals

As for several years it was quite evident that hospitals normally ignore the importance of healthy sleep and the power of every hour of sleep for the child's healing process. Sleep is crucial for recovery and well‐being in hospitalised children, but it is often disrupted due to environmental factors, medical interventions, and the child's illness. Noise from medical equipment, staff activities, and room‐sharing disrupt sleep. Excessive light, especially at night, interferes with the child's circadian rhythm. Further, frequent vital sign checks, medication administration, and diagnostic procedures often occur during night‐time, fragmenting sleep. It is also important to note that pain, anxiety, and discomfort from medical conditions or treatments reduce sleep quality. On the other hand, sleep has a fundamental role in clinical outcomes: better sleep is associated with faster recovery and shorter hospital stays and improving sleep reduces anxiety and improves cooperation during treatment. The interventions to promote children's sleep in hospitals should aim at noise reduction implementing quiet hours and using soundproof materials and controlling light exposure with dim lights at night and ensuring exposure to natural light during the day to support circadian rhythms. Furthermore, it is crucial to minimise night‐time disturbances, to ensure adequate pain relief to facilitate restful sleep and provide sleep aids like blankets or stuffed animals to reduce anxiety. Finally, allowing parents to stay overnight provides emotional comfort and can improve sleep quality. Sleep‐friendly hospitals might include not only the child's sleep but also further aspects such as room conditions, lightening, staff training, and also recommendations for the staff regarding their own sleep—especially concerning shifts and the impact on sleep and everyday life. Another related paediatric population that has been largely overlooked is children in palliative care settings, whose remaining quality of life (as well as that of their caregivers) may be significantly negatively impacted by sleeplessness or daytime somnolence for a wide variety of reasons (e.g., pain, medication, anxiety), and who could benefit from awareness of these issues and implementation of simple strategies (Mercante et al., 2024).

### 3.5 Clear recommendations for families

The prevalence of sleep issues among young children affects a diverse range of families, highlighting the necessity for tailored recommendations addressing paediatric sleep problems, particularly for fatigued parents (Schlarb, Schneider, & Quante, 2024). Such recommendations should be utilised by professionals in the field, including paediatricians, psychologists, and nurses, and subsequently disseminated to parents. Furthermore, it is essential that these guidelines prioritise user‐centred design, incorporating participatory development when feasible to enhance user‐friendliness and acceptance. Fortunately, many organisations and associations, too many to mention, are endeavouring to supply their guidelines and recommendations (American Academy of Sleep Medicine, IPSA, American Academy of Paediatrics, Raising Children's Network Australia, HealthDirect, Health Canada). However, there is a further pressing need for varied and age‐specific recommendations, particularly in contexts such as kindergarten environments, school settings, and among vulnerable populations such as foster children and refugees (Touchette et al., 2023), especially in light of challenges posed by war and natural disasters.

### 3.6 The ChildRight2Sleep (CR2S)

To enhance the importance of sleep a group of researchers and clinicians initiated the CR2S movement with the goal to provide not only healthcare professionals, but also the patient and the patient's family with timely primary or secondary preventive measures, knowledge of sleep medicine and psychology in the cultural concept or ecological model of the community. The main message of the CR2S is ‘Sleep is Prevention! Sleep is Primary, Secondary and Tertiary Prevention!’, to create the setting for early recognition of sleep disturbances and disorders is the main goal of this initiative. In this context, scientific societies for sleep medicine and sleep research, have a special, moderating role to play—to take up the content that can already be implemented by the general practitioner and/or by frontline workers, such as public health nurses and/or social workers and employees of the psychosocial services, and to harmonise it with the specialist societies for allowing a transdisciplinary and transdiagnostic observation based unselective screening approach (Ipsiroglu, Klösch, Stein, et al., 2024). Sleep as an essential part of development and health is stated as a necessity to be addressed in medical education and diagnostic procedures (Ipsiroglu, Klösch, Stein, et al., 2024). Besides those points, the initiative also wants to encourage young researchers and clinicians to engage in sleep medicine and science. Future developments might also address children in schools, teachers, parents, and persons with political responsibility.

## 4 EXOSYSTEM FACTORS

### 4.1 Child sleep in public communication

In the discourse surrounding paediatric sleep within the media, it is imperative to deliver clear, evidence‐based, and empathetic recommendations that resonate with parents and caregivers. The salient messages for media communication should emphasise the significance of sleep in fostering a child's physical health, emotional regulation, cognitive development, and overall maturation. Furthermore, it is essential to underscore the potential repercussions of sleep deprivation, which may manifest as behavioural disturbances, attentional deficits, and compromised immune function.

Comprehensive information should be presented regarding age‐appropriate sleep duration recommendations for various developmental stages, alongside strategies for promoting healthy sleep practices guided by principles of sleep hygiene. Additionally, it is pertinent to provide guidance for addressing prevalent sleep challenges, including bedtime resistance and nocturnal awakenings, while accentuating the role of parents as facilitators of change.

Moreover, the identification of ‘red flags’ indicative of sleep disorders, such as OSA, parasomnias, excessive daytime sleepiness, or sleep‐related movement disorders, should be incorporated into the communication framework.

These critical messages can be disseminated through social networks (Instagram, Facebook, Twitter, TikTok, etc.) and diverse mediums including blogs, infographics, video presentations, podcasts, and interactive live events or webinars featuring sleep experts. The media possesses an instrumental capacity to advocate for healthier sleep practices among children and their families. As professionals in the field, we bear both the opportunity and the obligation to elucidate the crucial importance of sleep in promoting the overall well‐being of children.

### 4.2 Prevention and school training – Sleep education at all levels

Preventive measures and educational campaigns to prevent sleep disorders in children will play a central role. Therefore raising awareness about the importance of healthy sleep in further settings is necessary (Blunden et al., 2012).

1. **Books:** to prevent common sleep problems, such as insomnia symptoms or coping with nightmares, more adequate age‐oriented books and bedtime stories are needed. Most of these books up to date include unhealthy sleep behaviour or strategies (Schlarb et al., 2020). However, future programmes might support children with age‐oriented healthy sleep behaviour strategies supported or introduced with their beloved animal thanks to artificial intelligence (AI).
2. **Video presentation:** in school children, videos on sleep might evoke more attention. A recently published study developed age‐oriented cartoon videos on sleep (Hassanin et al., 2023). The first video focused on the importance of sleep, while the second focused on different difficult sleep‐related scenarios, including recommendations on how to deal with sleep‐specific issues. The two clips are intended as prevention interventions, and such kind of prevention appeared to be successful in improving the awareness of the importance of sleep, including a longer sleep duration (Hassanin et al., 2023). Future studies might adapt such videos for other ages and comorbidities and investigate their long‐term effects.

### 4.3 Stepped care models (SCMs) for various sleep disorders in children and adolescents

The SCM, which is a staged system of intervention delivery that is organised from the least intensive to the most intensive, with the goal to match the needs of individuals with the correct intervention represents an approach to facilitating efficient and wide‐ranging provision of evidence‐based care to those children and adolescents with insomnia and other sleep disorders. The SCM not only includes diagnostics but also prevention possibilities, as well as information strategies concerning sleep health and low‐step prevention and age‐oriented material for different age groups and professions (teachers, healthcare providers, etc.). In the intervention section, besides online‐intervention and group intervention, personal face‐to‐face intervention in severe cases and comorbidities are the top. Such SCMs could help to plan treatments specifically tailored to a child's age, including influencing factors such as comorbidities (for insomnia in children in Germany see Schlarb, Schneider, & Quante, 2024, in press). Professional education of various health‐related professions such as paediatricians, psychologists, and nurses is enclosed in the SCM, along with digital medicine and face‐to‐face group interventions, single treatment or specialised interventions. Studies implementing strategic treatment options for paediatric insomnia as has been conducted for adults (Vincent & Walsh, 2013) might be a further step.

### 4.4 Medication

There are a number of steps that could be undertaken to improve the appropriate use of sleep medications in children. First, it should be noted that there are NO sleep medications approved for use by the United States Food and Drug Administration for children with insomnia apart from a controlled‐release melatonin for insomnia in children with autism or Smith–Magenis syndrome; thus, all use is ‘off label’ and largely without any paediatric clinical trial data regarding safety and efficacy. Pharmaceutical companies manufacturing these drugs need to be held accountable for conducting appropriate trials and publishing the results. Second, the variability in access to melatonin for children across global regions needs to be addressed. The variability in actual content of melatonin in OTC preparations available in the United States has been well‐demonstrated (Cohen et al., 2023; Erland & Saxena, 2017) and poses a potential health risk, including for families outside of the United States who order United States‐based melatonin products online. Third, additional independently funded studies need to be conducted in understudied paediatric populations (such as with typically‐developing children and melatonin); and fourth, educational materials for caregivers, healthcare professionals and other stakeholders such as one recently published in *Sleep Medicine* on melatonin in neurodiverse children (Edemann‐Callesen et al., 2023; Händel et al., 2023) need to be developed and disseminated to improve clinical practice Internationally, exogenous melatonin usage is increasingly common in children with sleep disturbance and indeed is one of the most frequently dispensed supplements for the treatment of sleep‐related disorders in children. Melatonin can aid with sleep/wake rhythm disturbances and significantly reduce sleep latency in individuals with sleep onset insomnia (Bruni et al., 2015; Waldron et al., 2016).

### 4.5 Special populations and populations with special needs

The same does not fit for all, therefore special adapted sleep information and interventions are needed.

1. University students: as university students are often late‐adolescents or early adulthood persons, they are at increased risk of both mental health issues, and sleep problems (Regli et al., 2024). Besides study‐related strains such as examinations and study‐related teamwork, further stressors might be additional job engagement or living in shared households. Given this, specifically tailored short‐term interventions might improve students’ mental and sleep‐related well‐being.
2. Neurodevelopmental disabilities: in future, increased awareness of the importance of sleep in the context of children with neurodiverse conditions might arise (Bruni et al., 2025). To date, it appears that both parents and their family doctors consider sleep problems as part of the diseased condition, while a specifically tailored sleep intervention might improve the child's psychological well‐being. Given this, more specific diagnostic tools and information programmes, as well as treatment options, should be developed for these populations (Spruyt et al., 2024).
3. Preterm birth: children born preterm might have long‐lasting deteriorations in sleep patterns, including a higher risk of OSA. Similarly to children with neurodiverse conditions, parents of children born preterm need more sleep‐related guidance both during the first years and later on. Future research might include parental training regarding sleep differences and possible upcoming health issues, information videos or clips and special trained paediatricians and nurses concerning this complex condition.

### 4.6 Personalised medicine

The future of paediatric sleep medicine will increasingly employ personalised approaches based on genetic, biological, environmental, cultural, and psychosocial factors. Genomic research could help identifying individual susceptibilities to specific sleep disorders and develop tailored treatments (Kocevska et al., 2024). Genome‐wide association studies have demonstrated that many significant genetic factors are associated with sleep‐related traits. Substantial genetic correlations have been reported between paediatric insomnia and psychiatric disorders suggesting a possible polygenic inheritance. Although environmental factors play an important role in the development of problematic sleep, genetic predisposition determines a considerable portion of the interindividual variability in sleep patterns (Kocevska et al., 2021, 2024). An example of personalised medicine could be paediatric insomnia. The manifestations of chronic insomnia undergo age‐related changes with age‐related distinct clusters of clinical presentations suggesting the presence of various phenotypes. Enhanced comprehension of insomnia's manifestations across diverse developmental stages can facilitate accurate assessment and may provide a framework for precision‐based treatment options (Bruni et al., 2024; Siriwardhana et al., 2020). In addition, although the frontline treatment for OSA in children is adenotonsillectomy, this is not curative in all cases and other treatments such as mandibular advancement or continuous positive airway pressure may be more effective in cases where tonsils and adenoids are not enlarged or where craniofacial features increase the risk of OSA.

### 4.7 More interdisciplinary collaboration

Because sleep is related to various aspects of all physical and mental health, close collaboration between various kinds of healthcare professionals such as paediatricians, psychologists, sleep specialists, and other professionals will be crucial. Interdisciplinary teams can create more comprehensive screening approaches, diagnosis and treatment plans that address both medical and psychosocial aspects (Berger et al., 2021; Horger, 2021; Mindell et al., 2011; Sambale et al., 2024). In addition, also including children, adolescents, students or parents in the treatment development phase might be helpful generating user‐friendly and also high‐acceptable recommendations and treatment options.

### 4.8 Long‐term sleep research

One of the previous papers regarding the future development and research gap, which was discussed in a conference for paediatric sleep medicine clinicians (Owens & Mindell, 2006), mentioned that there is a need for long‐term epidemiological studies of sleep patterns and sleep disorders in children. Moreover, in the recent years, sleep research often implemented only single questions regarding sleep and no standardised or even diagnostic instruments concerning sleep and the impact of insufficient or disrupted sleep in long‐term studies. This should be changed.

## 5 MICROSYSTEM FACTORS

### 5.1 Media usage and sleep in childhood

During the last few years an increasing number of studies have shown that media use already negatively affects young children's sleep, although, almost by default, studies on the media–sleep link are more abundant for adolescents. For adolescents, it appears that the fear of being cut from the stream of information (nomophobia) is associated with mental stress (Abdoli et al., 2023; Kater & Schlarb, 2020). However, more fine‐grained analyses of media‐related factors that trigger and exacerbate poor sleep are critically needed. To make the case in point, socially induced alertness, but not blue light emission, impacted sleep quality among young adults (Bowler & Bourke, 2019). As such, beyond mechanistic and biologist approaches to explain children's and adolescents’ sleep, cognitive‐emotional appraisals should be introduced with much more emphasis. By contrast, e.g., sleep‐related computer games or apps might help to improve sleep. This type of research could also be expanded to children in crises (see the war section).

### 5.2 Parental behaviour/sleep training

Parental sleep training prior to birth of a child, e.g., included in a prenatal class, might help future parents to adapt to the disrupted sleep they will experience in the first months to years (Blunden et al., 2015). Parents should be made aware of the development of sleep that occurs in infants through the first year of life, so they are prepared and have the necessary expertise and strategies to deal with this. Furthermore, sleep and the development of sleep or changes in sleep during pregnancy including the increased risk of OSA or insomnia disorders might be taught in midwifery seminars or students of midwifery. In addition, the importance of not sleeping on the back, particularly in late pregnancy, as this significantly increases the risk of stillbirth. Therefore, sleep should be a part of general parental education training in the future. Such training might help to establish routines and rules that gives the child a feeling of safety and help them to relax.

### 5.3 Sleep education for nursery teachers/advisory offices

There is often a need for sleep education for nursery teachers and advisory offices. As families with children with sleep problems often contact such centres and ask for help, education of these key stake holders might be very helpful. Such education might be structured and formulated by specialists regarding sleep of children and be guided, e.g., by associations like IPSA (Spruyt et al., 2024).

### 5.4 Parents with mental health problems and parents with physical impairments

Future research might focus on parental issues and their relation to their child's sleep. Besides a possible genetic transmission between parents’ and children's psychological strain such as symptoms of anxiety, depression and insomnia, following Bandura's social learning theory (Bandura, 1971), parents also serve as a role model for behaviour and coping, in general, and for daytime and night‐time behaviour, specifically. As such, children learn from their parents how to cope with stress and sleep‐related issues.

### 5.5 Family sleep – The interaction of sleep problems and health in the family

As sleep occurs at home, family interactions and dynamics should be considered. Typical factors impacting favourably or unfavourably on children's sleep are parents’ quality of partnership (Kelly & El‐Sheikh, 2013), including separation and divorce, parents’ parenting style (Brand, Gerber, et al., 2009; Brand, Hatzinger, et al., 2009), along with the quality of the parent–child relationship over time (Bell & Belsky, 2008). For example, the partnership quality of the parents, if they are divorced or have a patchwork structure or other special situations (see also health problems of parents), parental specificities such as shift work or living conditions might play a role. Therefore, there is a further need for behavioural sleep medicine/sleep psychology approaches to offer a broad range of evidence‐based therapies to account for these and other psychosocial factors. Given that an adolescent child's sleep is bi‐directionally associated with the parent's sleep, this also holds true for the child's and parent's psychological functioning (Brand, Gerber, et al., 2009; Brand, Hatzinger, et al., 2009; Kalak et al., 2012). Variations in a child's sleep are influenced by family resources, including caregivers’ availability, financial stability, and access to basic necessities. Time spent in bed, a factor that society can modify and that shapes both the quantity and quality of sleep, plays a crucial role in the relationship between stressors and sleep outcomes in families (Alaribe et al., 2021).

### 5.6 Child‐oriented sleep education – Bedtime books

As mentioned above, in recent years some sleep‐related children's books and booklets have been developed. In the future, more age‐oriented and gender‐oriented books and other education possibilities should be generated. A first study concerning sleep stories with the help of AI might be a way for the future (Schlarb & Faber, 2024). However, there is definitively a substantive need for such a basic element in sleep education and sleep help for children and adolescents. In addition, further research and development might also include the participation of the children using adequate strategies to implement their position. In the future, storybooks should consider more thoroughly gender and personal aspects such as weight, height, shape and colour of hair and colour of eyes to make it easier for children to self‐identify with the story. In addition, sleep‐helping figures might be more animated than before as described below.

### 5.7 Interactive sleep learning through animations

Animations might offer a dynamic and visually appealing way to teach children about healthy sleep habits. Digital sleep training programs can utilise colourful characters, engaging storylines, and interactive elements to convey important information about sleep hygiene. By embedding educational content within entertaining narratives, children are more likely to retain the information and apply it to their own routines. The *Sleep Ninja* for adolescents is a sleep app based on CBT‐I in Australia helping adolescents to implement their sleep schedules, and bedroom practices shift from day to night and reflect their beliefs about sleep and other aspects (Werner‐Seidler et al., 2019). A program for children like *Training for Kinder mit Schlaf‐Störungen* (KiSS) treatment with ‘Kalimba the Leopard with magic sleep helping spots’ can be adapted as one innovative example of interactive learning through animations is the KiSS treatment (Schlarb, 2014). Such interactive programs could be included in computer games or digital bedtime rituals. In addition, already available sleep games such as *The Nighty Night* sleep game for young children should be scientifically evaluated; more specifically, *The Nighty Night* sleep game contains a bedtime situation saying good night to various animals by turning off the lights. A criteria catalogue to evaluate such sleep‐related apps and games has been developed (Schlarb et al., 2024); such information helps both parents and healthcare providers to evaluate the utility and efficacy of such apps or games.

### 5.8 Personalised sleep plans

Future digital sleep training programs might be able to leverage data from wearable devices or sleep‐tracking apps to create adapted and personalised sleep plans for both children and above all adolescents. These plans can be tailored to address modifications of individual sleep patterns due to puberty, and such plans may include also factors such as school starting times, holidays and cultural differences and special circumstances, and issues (e.g., for children with special needs or sleeping in a room with others), providing customised guidance and recommendations (with its unique needs). Animated feedback and progress tracking can help children visualise their improvements and stay motivated.

## 6 GENERAL FUTURE MODIFICATIONS AND DEVELOPMENTS

1. Digitalising and adapting existing measures (questionnaires and sleep logs): there are various questionnaires regarding sleep for different ages and sleep problems. However, some of these should be translated into various languages and adapted for different ages. Furthermore, the digitalisation of sleep questionnaires such as the CSHQ, SDSC, or the Children Sleep Comic (CSC), may represent a significant advancement in paediatric sleep diagnostics. By leveraging digital versions, such measures become more accessible, accurate, and user‐friendly for not only paediatricians and sleep experts but also for parents and children. Digital versions of these questionnaires could be accessed via smartphones, tablets, and computers, making it easier for parents and children to complete them at home or prior to their sleep specialist appointment. This convenience reduces the barriers to participation (especially if picture‐based diagnostic instruments are available), potentially leading to higher fill‐out responses, higher response rates for measurements after or on long‐term treatments and more comprehensive data collection.
2. Data analysis and interpretation: to date, often sleep technicians score paediatric PSG data manually, which is a very accurate, although also a time‐consuming process. However, also the automatic analysis provided by the different PSG systems is often inaccurate for children and often produces a scientifically unacceptable number of issues and errors. PSG and wearable sleep monitors utilise highly variable outcome measures making cross‐study comparisons difficult and highlighting the need for standardisation in outcome measures and sensor deployment methodologies (Takagi et al., 2024). In the future, age‐adapted AI algorithms may help to analyse PSG data with higher accuracy. Machine learning algorithms can process complex data from PSG and wearable devices, enhancing the understanding of sleep disorders and their underlying causes (Bandyopadhyay & Goldstein, 2023). Furthermore, AI might be able to detect subtle patterns in physiological data that human scorers miss. This could lead to an earlier detection of sleep disorders and the discovery of new sleep‐related conditions in children (Davenport et al., 2024). In addition, AI algorithms can automatically classify sleep stages of different paediatric populations, e.g., premature or newborn babies or children with fetal alcohol syndrome disorder, and those with various health issues using data from wearable devices or PSG, reducing the time and effort required for manual scoring.
3. Development of further unobstructive monitoring tools for sleep and support: AI‐based analyses of sleep data gathered from wearable devices, such as smartwatches or pyjamas, might be a comfortable and affordable alternative to more traditional actigraphy or sleep‐EEG‐based sleep studies for children. Further, devices to assess sound, noise and movements in a child's bedroom may help to quantify sleep disruptions such as snoring or sleep apnea in a user‐friendly way (e.g., smart baby biometric monitoring devices like Nanit). New approaches using, e.g., wireless vital sign monitoring with radar technology are promising (Arasteh et al., 2023). In the future, also technical systems might be developed such as AI‐based chatbots offering age‐appropriate support for families with various bedtime or sleep problems adapted to their special situations. Eventually, such tools may be able to track the child's age‐appropriate sleep habits, and to provide information and reminders for good sleep practices to the caretakers. A further step might be also the implementation of a warning system for when to consult a sleep specialist.
4. Adaptation of sleep masks for children: future technical developments may also include AI to adapt sleep masks. While not yet mainstream, there are some interesting developments and potential applications: these sleep masks utilise AI features EEG sensors to monitor brain wave activities. In addition, it also implements AI‐powered music generation: The AI analyses brain waves and generates personalised music or soundscapes designed to promote relaxation and sleep. Last, the mask may provide brain wave biofeedback based on brain wave activities, potentially helping to train the brain for better sleep. These developments might be helpful for adolescents.
5. Sleep training with AI: future digital sleep training platforms might be able to also include typical family factors such as having a pet, a family member's chronic illness, other siblings, living close to construction areas so as to facilitate parental involvement. With such adaptations, parents might be more motivated to follow the suggestions, and they might easily receive updates on their child's progress. Importantly, the implementation of parents in such developments might help to enhance the acceptance of this specific population.
6. Sleep Research: digital assistance improves data accuracy and data quality. Digital platforms not only enable automated reminders and prompts, but they also ensure that questionnaires such as sleep diaries are completed fully and on time. Such platforms might enhance the reliability of the collected data. In addition, questionnaires, along with additional and related questions can be easily adapted and permutated to reduce the monotony and to improve the overall quality and accuracy of the data above all in the context of mental health issues. In addition, real‐time data analysis could be performed. Responses as a part of treatment (feedback) could be immediately provided. Lastly, digital platforms might offer the opportunity to present questionnaires in more appealing and interactive formats. To illustrate, the CSC (Schwerdtle et al., 2016) with its pictures could utilise multimedia elements, such as animations and gamified content, to make it more joyful to watch and to complete forms. In doing so, both the child's focus might remain constant, and the accuracy of the data might be more reliable. Further, the diagnostic comic style might be also transferred into intervention methods like sleep education kits or sleep training. Besides the diagnostics prior to beginning the treatment, the easy and regular updates of the questionnaires implemented in the digital sleep tools could facilitate the longitudinal tracking of sleep patterns. Over time, such an accurate data gathering could provide valuable insights into whether and to what extent a child's sleep habits evolve over time, thus enabling more precise and customised interventions (see personalised medicine).
*Predictive Modelling*: based on the above‐mentioned factors AI may be able to provide a combination of genetic, environmental, behavioural, and family‐related factors. As already shown in other disciplines, AI can be used to develop predictive models to identify children at risk of sleep disorders (especially in young children or even in pregnancy), and such an early identification may enable proactive interventions, potentially preventing the onset or worsening of sleep problems.
*Research Collaboration and dissemination*: AI can facilitate collaboration among researchers by summarising recent studies, generating hypotheses, and suggesting experimental designs. It could also assist in disseminating research findings to a broader audience, including clinicians, children and parents, but also teachers, or caretakers and policymakers. In addition, AI could also be included in the sleep education of professionals and in research as a current study has shown (Kim et al., 2024).

## 7 CONCLUSION

Over the last 50 years, paediatric sleep research has achieved much. Information about paediatric sleep is widely available and social media as well as healthcare professionals are more aware than ever that sleep plays a significant role in human health. However, several topics Owens and Mindell addressed nearly 20 years ago are still relevant today (Reynolds et al., 2023). Long‐term studies are needed, sleep‐measurements upgraded, and the link between various facets of paediatric sleep and their outcome (also long‐term outcome) concerning mental health and also physical health is still of interest. The development of clinical standards of practice and the need for sleep education for various healthcare providers is an ongoing process (Spruyt et al., 2024). As was stated by the American Academy of Sleep Medicine in 2021 (Ramar et al., 2021), sleep is essential to health; this is correct not only for adults but is even more important in children when sleep is at a lifetime maximum. Therefore, the future of paediatric sleep medicine holds promising developments and challenges.

Chronosystem factors and the effects of global warming on children's sleep need to be more thoroughly investigated and integrated into treatment strategies. In addition, crises such as natural disasters or man‐made crises such as war have to be taken into account regarding the child's sleep.

Macrosystem factors such as professional education, the CR2S initiative, education of paediatric sleep coaches, cultural differences and psychosocial factors will play a key role. Sleep‐friendly hospitals and precise recommendations for specific populations are facets that should be more developed in future.

Exosystem factors, such as the importance of paediatric sleep concerning all facets of health and development, with age‐appropriate recommendations, should be more widely available in the media. In addition, prevention of sleep problems and sleep education should be implemented at all levels of health professional education, as this may offer new challenges and changes in children's sleep (Spruyt et al., 2024). SCMs, not only for insomnia in childhood, but also for other disorders will be of importance. Including developments of technology and personalised approaches will help to implement them more interestingly. However, as medication is also an important issue concerning sleep treatment, further developments might be essential.

Future research might also focus more on populations with special needs, as these most often are not included in research studies. However, personalised medicine might include such aspects as further facets like genetics. All in all, more interdisciplinary cooperation is needed, inviting a broader range of professionals but also children or adolescents and their parents, which might be of profit for the future. Last but not least, more long‐term projects are needed, especially as the development of children is core for their future.

Regarding microsystem factors, media usage and sleep is becoming more and more important. We know a lot, but there is a need for further research. For parents, information on sleep and the development of sleep should be included in regular pre‐ and anti‐natal education programmes. In line with this, sleep education for nursery teachers and other professionals is a future task. In addition, the research might focus more on parents with various health problems and places more in the centre that children's sleep also means family sleep and family sleep affects the child's sleep. Last but not least, age‐oriented sleep education and sleep help might be a very important development in the upcoming years. Some bedtime books are already published but we need more sleep books according to age and topic for children and adolescents. Along with this, interactive sleep learning or training might include animations in the future, so that learning how to sleep healthy and well is amazing.

Despite those factors based on the system of Bronfenbrenner, further facets might be of interest as digitalisation of measures, data analysis and interpretation with AI, automated sleep staging, development of non‐invasive monitoring, and adaptation of sleep masks for children and their special needs or AI‐based training and the implementation of AI in future sleep research.

## AUTHOR CONTRIBUTIONS

**Angelika A. Schlarb:** Conceptualization; writing – original draft; visualization; methodology; project administration. **Sarah Blunden:** Writing – review and editing. **Serge Brand:** Writing – review and editing. **Olivero Bruni:** Writing – review and editing. **Penny Corkum:** Writing – review and editing. **Rosemary S. C. Horne:** Writing – review and editing. **Osman S. Ipsiroglu:** Writing – review and editing. **Mirja Quante:** Writing – review and editing. **Karen Spruyt:** Writing – review and editing. **Judith Owens:** Writing – review and editing.

## FUNDING INFORMATION

This research received no external funding.

## CONFLICT OF INTEREST STATEMENT

All authors have no known conflict of interest to disclose.

## Data Availability

Data sharing not applicable to this article as no datasets were generated or analysed during the current study.
